# Contribution of Ultrasound in Current Practice for Managing Juvenile Idiopathic Arthritis

**DOI:** 10.3390/jcm12010091

**Published:** 2022-12-22

**Authors:** Charlotte Borocco, Federica Anselmi, Linda Rossi-Semerano

**Affiliations:** 1 Department of Paediatric Rheumatology, National Reference Centre for Auto-Inflammatory Diseases and Amyloidosis of Inflammatory Origin (CEREMAIA), Bicêtre Hospital (AP-HP), 94270 Le Kremlin-Bicêtre, France; 2 Unit of Paediatric Rheumatology, Department of Translational Medicine, Section of Paediatrics, University of Naples Federico II, 80138 Naples, Italy

**Keywords:** ultrasonography, juvenile idiopathic arthritis, peculiarities, enthesitis, reliability, sensitivity, application, gestures, follow-up, treat-to-target

## Abstract

The interest and application of musculoskeletal ultrasound (MSUS) in juvenile idiopathic arthritis (JIA) are increasing. Numerous studies have shown that MSUS is more sensitive than clinical examination for detecting subclinical synovitis. MSUS is a well-accepted tool, easily accessible and non-irradiating. Therefore, it is a useful technique throughout JIA management. In the diagnostic work-up, MSUS allows for better characterizing the inflammatory involvement. It helps to define the disease extension, improving the classification of patients into JIA subtypes. Moreover, it is an essential tool for guiding intra-articular and peritendinous procedures. Finally, during the follow-up, in detecting subclinical disease activity, MSUS can be helpful in therapeutic decision-making. Because of several peculiarities related to the growing skeleton, the MSUS standards defined for adults do not apply to children. During the last decade, many teams have made large efforts to define normal and pathological US features in children in different age groups, which should be considered during the US examination. This review describes the specificities of MSUS in children, its applications in clinical practice, and its integration into the new JIA treat-to-target therapeutic approach.

## 1. Introduction

Juvenile idiopathic arthritis (JIA) is the most common chronic rheumatic disease in childhood, with an estimated prevalence of 3.8 to 400 per 100,000 children [1]. It includes a heterogeneous group of inflammatory arthritis of unknown etiology with a minimum duration of 6 weeks and onset before the age of 16. According to current International League of Associations for Rheumatology (ILAR) criteria, JIA is classified into 7 subtypes based on the number of joints involved, the presence or absence of extra-articular involvement, and the involvement of additional biological markers, such as an anti-nuclear antibody, rheumatoid factors, and HLA–B27 [2]. All subtypes are characterized by chronic synovitis, leading, if not adequately recognized and treated, to significant structural joint changes and functional disability. Despite advances in the knowledge of clinical features of JIA, the diagnostic delay is still long [3]. Nevertheless, early diagnosis and definition of the JIA subtype are crucial to initiating an appropriate and prompt treatment: the longer the delay in diagnosis, the more active the disease remains, with the risk of poor long-term outcomes [4].

Musculoskeletal US (MSUS) is a validated and reproducible tool for the diagnosis and follow-up of adults with chronic rheumatic diseases [5,6,7,8]. Its interest and applications as a key diagnostic and monitoring tool in pediatric rheumatology are also increasing [9,10,11]. Indeed, it is a well-accepted, easily accessible, non-irradiating, and inexpensive imaging technique that allows for performing a dynamic examination of several joints during the same session (Table 1). MSUS is particularly suitable for young children, who often need sedation for long imaging examinations such as MRI, which, unlike US, requires immobility.

MSUS is a useful tool at different stages in the management of JIA. In the diagnostic work-up, still based on clinical examination, MSUS allows for confirming the diagnosis in case of doubt and better characterizing the inflammatory involvement of the joint structures, ultimately helping to define the disease extension. It is an essential tool in guiding intra-articular and peritendinous procedures. Finally, during follow-up, because US is more sensitive than clinical examination for detecting subclinical synovitis, its results are helpful in therapeutic decision-making. It is also more sensitive than conventional radiography in the early detection of structural damage. High-resolution ultrasound (US) devices have been recently developed, improving the resolution of B-mode and Doppler mode images, thus encouraging the use of MSUS in clinical practice.

## 2. Pediatric Population US Features

**Figure 1:**
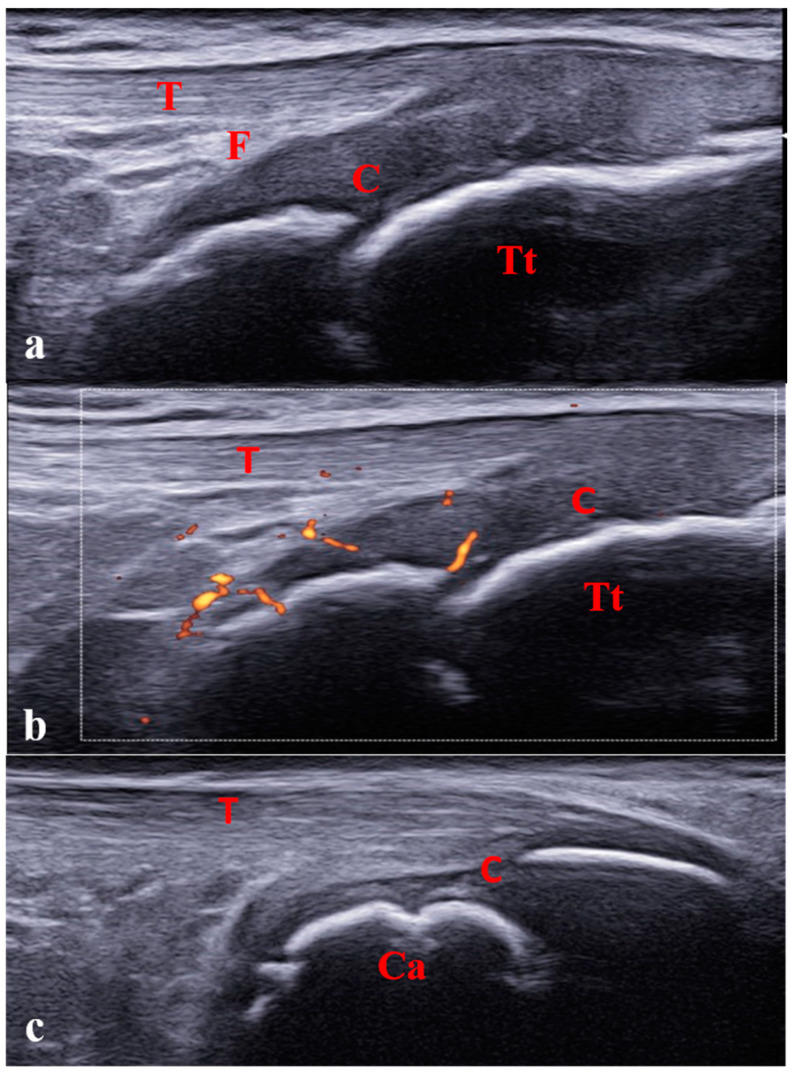
Pediatric ultrasound peculiarities. Increased ratio of cartilage to bone (**a**) and physiological cartilage vascularization (**b**) in a 6-year-old girl. (**c**) Physiological irregularity of calcaneus bone surface owing to the presence of ossification centers in a 10-year-old boy. C: cartilage; Tt: tibial tuberosity; Ca: calcaneus; T: tendon; F: fat.

As for its increased use in adult rheumatoid arthritis, the use of MSUS in pediatric rheumatology has widely expanded over the past 15 years. Nevertheless, because of several features related to the growing skeleton of children, the adult US definitions and scoring systems do not apply to pediatric patients. The main differences between adult and pediatric US features are the increased ratio of cartilage to bone, the physiological cartilage vascularization, and the presence of ossification centers and growth plates (Figure 1). In fact, children have thicker cartilage than adults, which becomes progressively thinner during growth [12]. Cartilage thickness differs according to gender, with boys showing thicker cartilage than girls. Reference measures of cartilage thickness according to gender and age have been described [13,14,15]. Because of the abundance of cartilage in young children, discriminating between synovial inflammation and the contiguous unossified cartilage is challenging, both having a hypoechoic appearance. Dynamic US examination allows for accurately distinguishing pathological findings from physiological cartilage, which is not compressible during joint movement or transducer pressure.

The articular surfaces aspect is physiologically irregular owing to the presence of the ossification centers, and distinguishing between the physiological appearance and pathological cortical irregularities or erosions might be difficult (Figure 1c).

**Figure 2:**
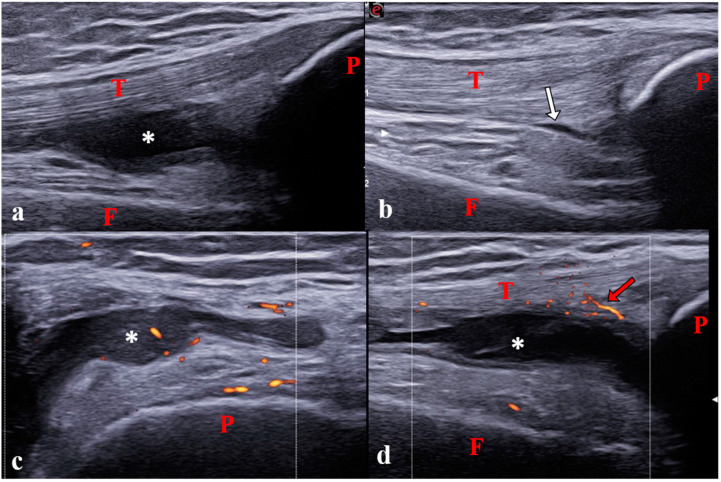
Synovitis of the knee joint in a 12-year-old girl with polyarticular juvenile idiopathic arthritis. (**a**) Longitudinal suprapatellar scan showing synovitis in the suprapatellar recess on B-mode (grade 2). (**b**) Physiological aspect of the contralateral joint on B-mode. (**c**) Transverse lateral parapatellar scan showing pathological vascularization (power Doppler grade 1). (**d**) Longitudinal suprapatellar scan showing vascularization outside the synovial hypertrophy (power Doppler grade 0). Note that the transverse scan is more sensitive for detecting pathological vascularisation inside synovitis than the longitudinal scan [18]. P: patella; T: tendon; F: femur; *: Synovitis; white arrow: small physiological amount of intra-articular fluid; Red arrows: physiological vascularization.

Another pediatric feature is the presence of feeding vessels within the growth plates, particularly in the epiphyseal cartilages, which is common in young children and progressively disappears with growth [13]. This physiological vascularization may be misinterpreted as a pathological Doppler signal corresponding to active synovitis (Figure 2).

Concerning entheses, defined as the area where tendons, ligaments, and joint capsules are inserted into the bone, children show the same fibrillar structure as adults. In contrast to cartilage, enthesis thickness progressively increases during growth [16,17].

Therefore, owing to these distinctive pediatric features and to support the growing role and use of MSUS in pediatric rheumatology, normative data and international consensus for the MSUS definitions and scanning techniques in healthy children were needed. The OMERACT pediatric US task force first proposed a set of consensus-based definitions concerning the normal joint components in healthy children using B-mode and Doppler mode [16,18,19]. It also proposed a standardized MSUS protocol describing the correct patient and probe position for examining the knee, ankle, wrist, and metacarpophalangeal (MCP) 2 joint [18]. Of note, other studies have described age-related standard measures of the hip, knee, wrist, elbow, and shoulder [20,21,22,23,24]. Furthermore, other authors focused on pediatric entheses, especially in lower limbs, describing the MSUS procedure, the structural components, and the physiological vascularization of the entheses according to different age groups [17,25,26].

For the best resolution and because of the smaller size of joints, the use of a high-frequency linear transducer is recommended in children (up to 22 MHz for superficial and small joints). However, according to the size and depth of different joints, the frequency should be lowered, especially in older children. Moreover, to improve MSUS examination, one must achieve adequate contact with the transducer. Therefore, especially for regions with small sizes and/or curved contours (e.g., ankle in a young child), one should use an abundant amount of gel. Finally, the exam must be performed under optimal conditions, with children being distracted and feeling comfortable.

**Figure 3:**
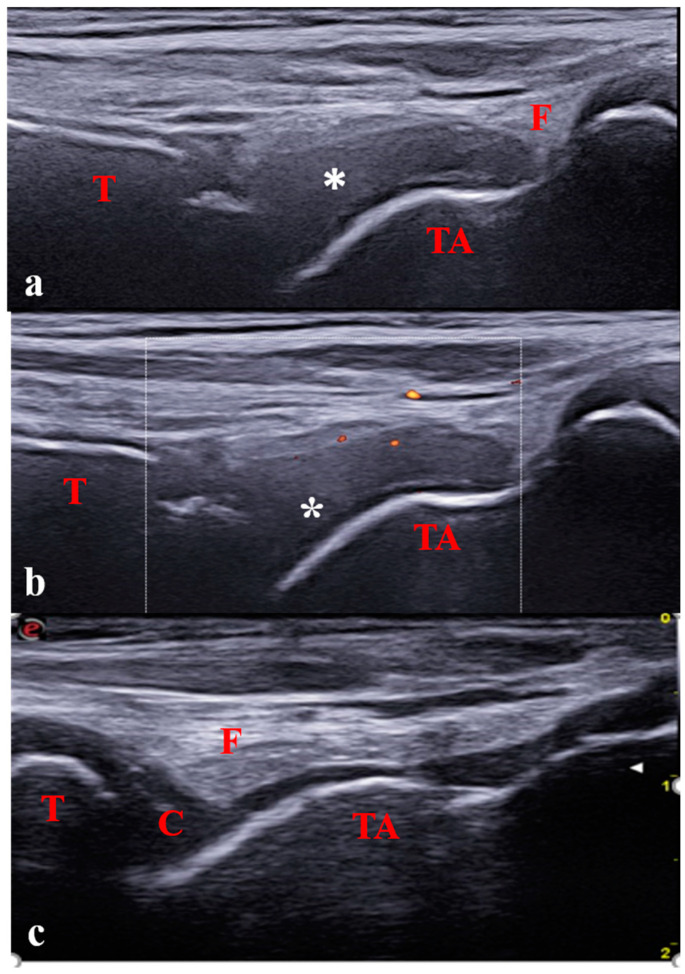
Longitudinal anterior scan of the tibiotalar joint in a 7-year-old with oligoarticular JIA; (**a**,**b**) Severe synovitis (B-mode grade 3) with minimal Doppler activity (grade 1). (**c**) Physiological aspect of the contralateral joint. C: cartilage; T: tibia; TA: talus; F: fat; * Synovitis.

Recent studies provided definitions and scoring systems of synovitis in children according to the extent of inflammation (Figure 2) [27,28]. For instance, today, a positive Doppler signal is not considered mandatory for diagnosing synovitis in children. Only the presence of positive Doppler signals within the synovial hypertrophy is considered a pathological finding suggesting synovitis. Thus, synovitis is defined as the combination of B-mode findings (synovial effusion and/or synovial hypertrophy) with or without Doppler signals (Figure 3). Concerning B-mode findings, synovial effusion is defined as an abnormal intra-articular fluid that is anechoic or hypoechoic and is displaceable and synovial hypertrophy as an intra-articular material that is hypoechoic and non-displaceable.

**Figure 4:**
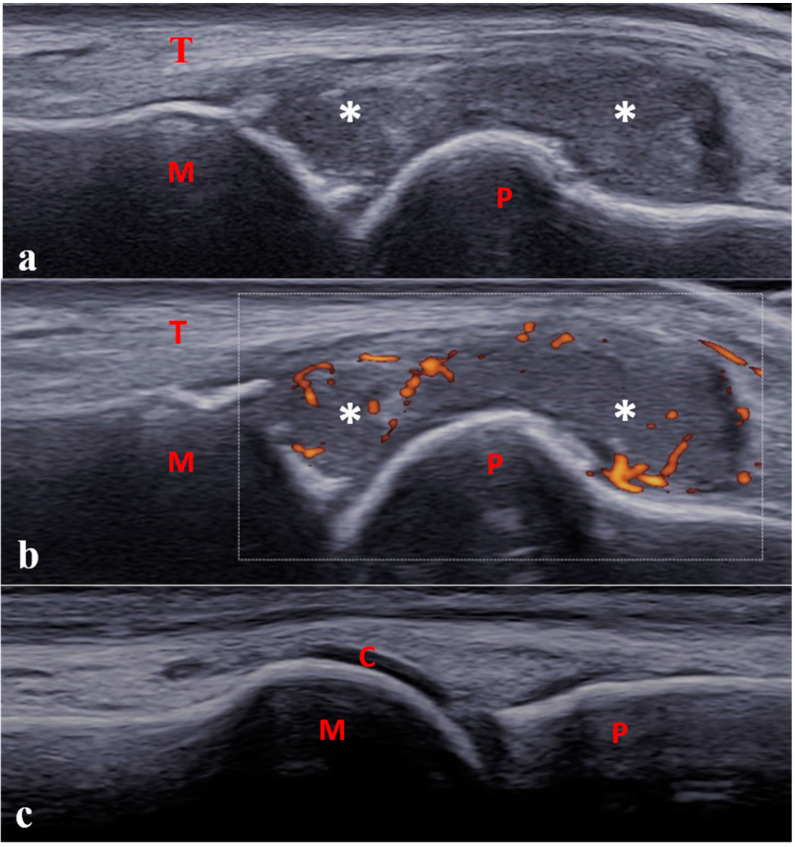
Longitudinal dorsal scan of one metatarsophalangeal joint in a 13-year-old boy with enthesitis-related arthritis. (**a**,**b**) Severe synovitis (B-mode grade 3) with moderate power Doppler activity (<30% of the synovial hypertrophy surface, grade 2). (**c**) Physiological aspect of the contra-lateral joint. C: cartilage; M: metatarsal; P: proximal phalanx; T: tendon; * Synovitis.

Concerning Doppler mode, power or color Doppler can be used, with the recommended following setting: pulse repetition frequency (PRF) range from 500 to 750 Hz depending on the examined joint and used device; gain set just below the level of appearance of color noise below the cortical bone surface, low-wall filters. Doppler scoring is defined differently in children than in adults [28]. Grade 1 is defined by the detection of up to 3 single Doppler signals within the area of synovial hypertrophy (Figure 3). Grades 2 and 3 are defined by the detection of Doppler signals within ±30% of the area of synovial hypertrophy (Figure 4).

According to these findings, an atlas including an acquisition protocol for the main joints involved in JIA was recently developed [29]. Moreover, to address the peculiarities and limitations of MSUS in children, pediatric-specific courses have been recently proposed by the Pediatric Rheumatology European Society (PReS) and the European League Against Rheumatism (EULAR).

**Figure 5:**
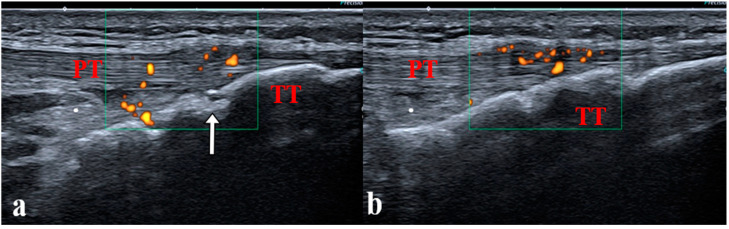
(**a**,**b**) Enthesitis of the distal patellar ligament in a 14-year-old boy with juvenile enthesitis-related arthritis. Longitudinal scan of the distal patellar ligament showing bilateral enthesitis. PT: patellar tendon; TT: tibial tuberosity; white arrow: secondary ossification center, incompletely ossified tibia.

**Figure 6:**
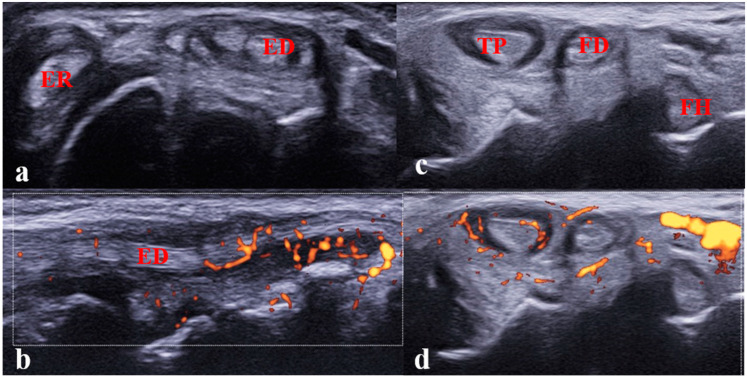
(**a**,**b**)**.** Tenosynovitis of the extensor digitorum and extensor carpi radialis tendons in a 12-year-old girl with JIA. (**a**) Transverse scan showing tenosynovitis on B-mode; (**b**) longitudinal scan showing abnormal vascularization on power Doppler mode. Performing the longitudinal scan (B-mode/Doppler mode) is mandatory to confirm the diagnosis of tenosynovitis. ED: extensor digitorum tendon; ER: extensor carpi radialis tendon; (**c**,**d**). Tenosynovitis of the tibialis poster tendon in an 8-year-old girl with JIA. Transverse scan of the medial compartment showing tenosynovitis.

To date, consensus definitions of tenosynovitis and enthesitis in children are still lacking; therefore, current definitions validated in adults are still applied to children [30,31]. Efforts to delineate pediatric definitions of tenosynovitis and enthesitis are in progress (Figure 5 and Figure 6) [32,33].

Finally, the reliability of US in detecting inflammatory lesions has been evaluated in several studies with good results, encouraging the application of these definitions and scoring systems in clinical practice [13,29,34,35].

## 3. Applications in Clinical Practice

### 3.1. Diagnosis

MSUS plays a key role in the diagnostic work-up of JIA. It is more sensitive in detecting articular inflammation than clinical examination alone [36,37,38,39]. In particular, the superiority of US for detecting synovitis compared to clinical examination has been demonstrated for certain joints, such as the ankle [40,41,42]. MSUS is also an important diagnostic tool in atypical clinical presentations, especially in young children who are often difficult to examine and do not express pain well. MSUS can also be helpful in patients with severe arthralgia. Indeed, some patients are in so much pain that they cannot be mobilized, thus impairing an adequate clinical examination. MSUS can then be used to assess the painful joints.

Moreover, MSUS is useful in characterizing the articular structures involved in inflammation, thus distinguishing between synovitis, tenosynovitis, or enthesitis [43]. US overall examination, including extra-articular compartments (bursae and tendons), is recommended. MSUS may explain up to 44.4% of positive clinical examinations without real joint involvement of the wrist and ankle [44].

Owing to its ability to detect subclinical synovitis, performing a US examination of several joints at the time of diagnosis allows for assessing the correct extension of JIA (e.g., number of joints involved), improving the baseline classification and, consequently, the therapeutic management.

MSUS is also a good tool for detecting enthesis abnormalities. The most commonly involved entheses on US examination in children with JIA are the Achilles tendon, quadricipital tendon, and distal insertion of the patellar ligament [45,46,47]. Clinically, enthesitis in children is generally investigated and detected by palpating a painful enthesis [2]. Early studies in children with enthesitis-related arthritis showed a poor correlation between clinical and MSUS examination; the latter was more sensitive and specific for detecting enthesitis. Nevertheless, a correlation was found between the presence of high-grade Doppler activity and clinical enthesitis [45,46,47]. As a reminder, at present, consensual definitions of enthesitis in children are still lacking.

As previously mentioned, MSUS allows for detecting and characterizing tenosynovitis. Tenosynovitis is not a pathognomonic feature of JIA and may be associated with other inflammatory diseases. For instance, a diagnosis of sarcoidosis, even at an early stage (e.g., Blau syndrome), can be evoked by the presence of a joint swelling on clinical examination, which corresponds to tenosynovitis on MSUS examination in most children [48]. Likewise, in adults with sarcoidosis, arthritis is often associated with tenosynovitis [49].

Also, the combination of synovitis and tenosynovitis (often subclinical), especially in adults, is a common presentation of systemic lupus erythematosus, so MSUS has a key role in the diagnostic confirmation [50,51].

Finally, US, combined with other imaging techniques, such as radiography and MRI, can be useful in the differential diagnosis, helping to distinguish JIA from arthritis of infectious origin, trauma, or chronic pain syndromes.

### 3.2. Treatment

Intra-articular corticosteroid injections (IACIs) are a widespread and validated treatment in JIA [52]. IACIs have historically been performed without MSUS guidance, referring only to the palpation of swollen joints and anatomical landmarks. However, owing to the potential side effects, such as subcutaneous atrophy and skin hypopigmentation, as well as the risk of procedure failure, increasing the precision of IACIs is crucial to improving effectiveness and safety. Because MSUS allows for real-time guidance of the needle and better control of the corticosteroid injection, US guidance of IACIs has been largely used in adult rheumatology but is now spreading to JIA.

Some deep or small joints are sometimes difficult to access with palpation alone and may be challenging to treat. This is the case for the midfoot, wrist, subtalar, and hip joints. Infiltration protocols have been established to improve the precision of IACIs in children [53,54,55].

The use of MSUS guidance is particularly recommended during the injection of tenosynovitis or synovial cysts. The MSUS precision allows for reaching and injecting corticosteroids into the peritendinous sheet, thus avoiding the potential rupture of the tendon deriving from an erroneous direct tendon injection. Of note, these injection techniques should be performed by US-trained physicians.

### 3.3. Follow-Up

As previously discussed, MSUS is more sensitive than clinical examination for detecting subclinical synovitis. However, to detect subclinical synovitis, one must perform an extended MSUS examination, which may be difficult in young children because of their poor compliance during long examinations. Thus, some authors focused on developing a minimal US joint assessment that can make the examination feasible while accurately detecting subclinical synovitis. Collado et al. showed a limited screening of 10 joints, including knees, ankles, wrists, elbows, and MCP 2 joints, correlated with the examination of 44 joints during the follow-up of JIA with at least 4 active joints [56]. Another study including polyarticular patients also suggested a reduced US joint score (MUSICAL score) based on 10 joints (knees, wrists, MCP joints 2 and 3, and ankles) [57]. We believe that a 10-joint US examination is feasible in routine practice. Nevertheless, these scores have not been validated in different JIA subtypes. Finally, a multicenter longitudinal study currently ongoing is evaluating the sensitivity and predictive value of a multi-biomarker panel, including clinical examination, ultrasound of 44 joints, and inflammatory biomarkers. It aims to establish a core set of minimally representative joint counts necessary to be assessed in everyday clinical practice [58].

During routine follow-ups of patients in clinical remission, the detection of subclinical US abnormalities has been suggested to be associated with a significant risk of relapse, especially in the case of positive Doppler signals [59,60]. Thus, identifying prognostic MSUS factors in JIA in clinical remission could play a key role in therapeutic management, leading to the continuation/intensification of the disease-modifying therapy in patients with sonographic abnormalities or, conversely, the discontinuation/reduction of therapy in patients without sonographic abnormalities.

Nevertheless, a study of adults with rheumatoid arthritis and a systematic MSUS follow-up over 2 years found no significant difference in clinical remission, radiographic progression, or quality of life compared to patients who were solely followed with clinical examination [61]. Finally, 2 other pediatric studies did not find any predictive value of MSUS on flares in patients in clinical remission [62,63]. Further studies are needed to validate the prognostic value of MSUS on the medium- to long-term outcome of JIA and the pertinence of systematic US follow-up combined with clinical examination. The use of the new pediatric definitions and scoring system in future studies will allow for standardizing the protocols by the use of shared outcome measures.

In recent years, the treat-to-target approach has spread in JIA management to achieve inactive disease as rapidly as possible and reduce the long-term consequences [64]. To date, clinical remission is defined according to Wallace criteria and/or the Juvenile Arthritis Disease Activity Score, which combines clinical and biological findings and parent/patient well-being. The response to disease-modifying anti-rheumatic drugs (biological or conventional) is generally assessed 3 months after their introduction, which is the minimum estimated time for seeing clinical and biological improvements [65]. However, owing to the high sensitivity of MSUS in revealing subclinical changes in joint structures, many studies of adults have reported the efficacy of MSUS in revealing early signs of therapeutic success [66]. Some studies of adults have shown that MSUS subclinical synovitis is correlated with interleukin 6 and S100 serum levels [67], the latter currently recognized as a new biomarker of disease activity and treatment response in JIA [68,69]. MSUS assessment in JIA patients at 4 weeks after IACI showed a reduction in synovial inflammation [70]. However, the presence itself of a residual synovitis also resulted in an increased risk of disease flare [59]. These results suggest that MSUS may be included in the treat-to-target approach in JIA, especially focusing on MSUS remission as a new treatment target. Indeed, if MSUS findings still favor disease activity 3 months after treatment introduction, a change in therapy could be considered for better long-term outcomes [71].

US follow-up of patients with JIA may also be useful for detecting structural damage. Conventional radiography is still recommended to identify erosions, especially in peripheral joints in patients with polyarticular JIA and a positive rheumatoid factor [72,73,74]. Nevertheless, it shows erosive changes late in the disease course, and repeating this examination increases the risk of radiation exposure. MSUS, with its ability to evaluate the joint in several planes, is a valuable tool for detecting erosions, with good inter- and intra-observer reproducibility [34]. However, assessment of bone changes in the growing skeleton can be challenging, especially in young children, because of physiological irregularities related to secondary ossification centers. Moreover, MSUS is not superior to conventional radiographs for detecting erosions in the wrist of JIA patients [75]. MRI seems more sensitive than US and conventional radiography for detecting structural lesions in JIA, especially when the diagnosis is recent.

Although its role in detecting bone erosions is still debated, MSUS has value in detecting cartilage damage, according to several studies. Indeed, in comparison to healthy controls, patients with JIA show decreased cartilage thickness, which may be an early sign of structural damage [76]. A recent study found thicker cartilage in unaffected than affected joints in JIA patients, suggesting that inflammation influences cartilage thickness [77]. Notably, cartilage is thinner in polyarticular and systemic JIA than in oligoarticular JIA.

## 4. Conclusions

The role of MSUS in JIA is definitely growing. To correctly interpret MSUS results and avoid misdiagnosis, knowledge of the particular sonographic features in children is still mandatory.

MSUS represents a key tool for confirming JIA, better classifying JIA subtypes, and characterizing articular and peri-articular inflammation. Intra-articular and peri-tendinous injections can be guided by MSUS, improving the accuracy of procedures and treatment efficacy. During JIA monitoring, the evaluation of treatment response by MSUS allows for detecting subclinical inflammation, which suggests a role for MSUS in the modern treat-to-target approach. Nevertheless, the type and number of joints to be routinely examined in clinical practice for accurately evaluating subclinical synovitis are still being evaluated.

MSUS may have a potential role in evaluating early damage, but the role of MRI should not be overlooked, especially for detecting bone erosions in recent-onset diseases. Further studies are needed to establish the predictive value of MSUS for clinical flare and overall long-term outcomes. Finally, data are needed to evaluate the value of MSUS as an outcome measure in clinical trials of ongoing and new therapies in patients with JIA.

## Figures and Tables

**Table 1:** Advantages, limitations, and peculiarities of musculoskeletal ultrasound in children with juvenile idiopathic arthritis.

Advantages	Limitations	Pediatric Peculiarities
Non-invasive (no need for sedation/contrast)	Operator and equipment dependency	Adaptation of probe frequency (usually high) according to age and size
Non-irradiating	Risk of misinterpretation of artefacts	Optimal comfort conditions to obtain children’s compliance
Repeatable	Acoustic shadowing from overlying bones	Increased cartilage/bone ratio, age, and sex-related cartilage thickness
Relatively inexpensive	Limited field of view (no access to whole joint space)	Presence of ossification centers and growth plates (irregular bone surfaces)
Bedside	Difficult to perform in case of severe joint limitation	Changes of US aspects with age
Short examination time (according to the number of joints assessed)		Physiological cartilage vascularization
High patient acceptability		
Dynamic examination		
Multiple simultaneous joints examinations, comparison with contralateral joint	No pediatric-specific validated protocols (number and type of joints)	
High-sensitivity for subclinical synovitis/enthesitis	No consensual definitions of enthesitis and tenosynovitis	Specific definition of synovitis (Doppler non-essential, pathological only if within synovial hypertrophy)
High-resolution soft tissue imaging (detection of inflammation of articular and peri-articular structures)	No validated predictive values of risk of flare	Specific Doppler scoring system
Early detection of structural damage (especially cartilage involvement)	Poor value in TMJ and axial skeleton assessment	
Ability to guide procedures	Need for long and specific operator training in pediatric patients Specific pediatric US courses not available in all Countries	

US: ultrasound; TMJ: temporomandibular joint.

## Data Availability

No new data were created.

## References

[1] Thierry S., Fautrel B., Lemelle I., Guillemin F. (2014). Prevalence and Incidence of Juvenile Idiopathic Arthritis: A Systematic Review. Jt. Bone Spine.

[2] Petty R.E., Southwood T.R., Manners P., Baum J., Glass D.N., Goldenberg J., He X., Maldonado-Cocco J., Orozco-Alcala J., Prieur A.-M. (2004). International League of Associations for Rheumatology Classification of Juvenile Idiopathic Arthritis: Second Revision, Edmonton, 2001. J. Rheumatol..

[3] Aoust L., Rossi-Semerano L., Koné-Paut I., Dusser P. (2017). Time to Diagnosis in Juvenile Idiopathic Arthritis: A French Perspective. Orphanet J. Rare Dis..

[4] Glerup M., Herlin T., Twilt M. (2017). Clinical Outcome and Long-Term Remission in JIA. Curr. Rheumatol. Rep..

[5] Ohrndorf S., Backhaus M. (2015). Pro Musculoskeletal Ultrasonography in Rheumatoid Arthritis. Clin. Exp. Rheumatol..

[6] Wakefield R.J., Goh E., Conaghan P.G., Karim Z., Emery P. (2003). Musculoskeletal Ultrasonography in Europe: Results of a Rheumatologist-Based Survey at a EULAR Meeting. Rheumatology.

[7] Backhaus M., Kamradt T., Sandrock D., Loreck D., Fritz J., Wolf K.J., Raber H., Hamm B., Burmester G.R., Bollow M. (1999). Arthritis of the Finger Joints: A Comprehensive Approach Comparing Conventional Radiography, Scintigraphy, Ultrasound, and Contrast-Enhanced Magnetic Resonance Imaging. Arthritis Rheum.

[8] Naredo E., Bonilla G., Gamero F., Uson J., Carmona L., Laffon A. (2005). Assessment of Inflammatory Activity in Rheumatoid Arthritis: A Comparative Study of Clinical Evaluation with Grey Scale and Power Doppler Ultrasonography. Ann. Rheum. Dis..

[9] Roth J. (2020). Emergence of Musculoskeletal Ultrasound Use in Pediatric Rheumatology. Curr. Rheumatol. Rep..

[10] Lanni S. (2021). The Recent Evolution of Ultrasound in Juvenile Idiopathic Arthritis. Clin. Exp. Rheumatol..

[11] Vega-Fernandez P., Ting T.V., Pratt L., Bacha C.M., Oberle E.J. (2022). Ultrasonography in Pediatric Rheumatology. Rheum. Dis. Clin. N. Am..

[12] Spannow A.H., Pfeiffer-Jensen M., Andersen N.T., Herlin T., Stenbøg E. (2010). Ultrasonographic Measurements of Joint Cartilage Thickness in Healthy Children: Age- and Sex-Related Standard Reference Values. J. Rheumatol..

[13] Windschall D., Collado P., Vojinovic J., Magni-Manzoni S., Balint P., Bruyn G.A.W., Hernandez-Diaz C., Nieto J.C., Ravagnani V., Tzaribachev N. (2020). Age-Related Vascularization and Ossification of Joints in Children: An International Pilot Study to Test Multiobserver Ultrasound Reliability. Arthritis Care Res..

[14] Jousse-Joulin S., Cangemi C., Alavi Z., Gerard S., Nonent M., Bressollette L., De Parscau L., Devauchelle-Pensec V., Saraux A. (2018). Normal Sonoanatomy of Small Joints in Healthy Children: Changes in Cartilage and Vascularisation According to Age and Gender. Clin. Exp. Rheumatol..

[15] Samanta M., Mitra S., Samui P.P., Mondal R.K., Hazra A., Sabui T.K. (2018). Evaluation of Joint Cartilage Thickness in Healthy Children by Ultrasound: An Experience from a Developing Nation. Int. J. Rheum. Dis..

[16] Roth J., Jousse-Joulin S., Magni-Manzoni S., Rodriguez A., Tzaribachev N., Iagnocco A., Naredo E., D’Agostino M.A., Collado P. (2015). Outcome Measures in Rheumatology Ultrasound Group Definitions for the Sonographic Features of Joints in Healthy Children. Arthritis Care Res..

[17] Jousse-Joulin S., Cangemi C., Gerard S., Gestin S., Bressollette L., de Parscau L., Devauchelle-Pensec V., Saraux A. (2015). Normal Sonoanatomy of the Paediatric Entheses Including Echostructure and Vascularisation Changes during Growth. Eur. Radiol..

[18] Collado P., Vojinovic J., Nieto J.C., Windschall D., Magni-Manzoni S., Bruyn G.A.W., Iagnocco A., D’agostino M.A., Naredo E. (2016). Omeract Ultrasound Pediatric Group toward Standardized Musculoskeletal Ultrasound in Pediatric Rheumatology: Normal Age-Related Ultrasound Findings. Arthritis Care Res..

[19] Collado P., Windschall D., Vojinovic J., Magni-Manzoni S., Balint P., Bruyn G.A.W., Hernandez-Diaz C., Nieto J.C., Ravagnani V., Tzaribachev N. (2018). Amendment of the OMERACT Ultrasound Definitions of Joints’ Features in Healthy Children When Using the DOPPLER Technique. Pediatr. Rheumatol. Online J..

[20] Trauzeddel R.F., Lehmann H., Windschall D., Ganser G., Berendes R., Haller M., Krumrey-Langkammerer M., Palm-Beden K., Nimtz-Talaska A., Nirschl C. (2017). Age-Dependent Arthrosonographic Reference Values of the Hip Joint in Healthy Children and Adolescents—A Cross-Sectional Multicenter Ultrasound Study. Pediatr. Radiol..

[21] Windschall D., Trauzeddel R., Haller M., Krumrey-Langkammerer M., Nimtz-Talaska A., Berendes R., Ganser G., Nirschl C., Schoof P., Trauzeddel R.F. (2016). Pediatric Musculoskeletal Ultrasound: Age- and Sex-Related Normal B-Mode Findings of the Knee. Rheumatol. Int..

[22] Rosendahl K., Bruserud I.S., Oehme N., Júlíusson P.B., de Horatio L.T., Müller L.-S.O., Magni-Manzoni S. (2018). Normative Ultrasound References for the Paediatric Wrist; Dorsal Soft Tissues. RMD Open.

[23] Trauzeddel R., Lehman H., Trauzeddel R.F., Haller M., Palm-Beden K., Ganser G., Berendes R., Nirschl C., Nimtz-Talaska A., Krumrey-Langkammerer M. (2019). Age Dependent Ultrasound B-Mode Findings of the Elbow Joint in Healthy Children and Adolescents. Rheumatol. Int..

[24] Trauzeddel R., Windschall D., Trauzeddel R.F., Nirschl C., Ganser G., Palm-Beden K., Berendes R., Haller M., Krumrey-Langkammerer M., Nimtz-Talaska A. (2017). Arthrosonographic Reference Values of the Shoulder Joint in Healthy Children and Adolescents: A Cross-Sectional Multicentre Ultrasound Study. Klin. Padiatr..

[25] Chauvin N.A., Ho-Fung V., Jaramillo D., Edgar J.C., Weiss P.F. (2015). Ultrasound of the Joints and Entheses in Healthy Children. Pediatr. Radiol..

[26] Roth J., Stinson S.E., Chan J., Barrowman N., Di Geso L. (2019). Differential Pattern of Doppler Signals at Lower-Extremity Entheses of Healthy Children. Pediatr. Radiol..

[27] Roth J., Ravagnani V., Backhaus M., Balint P., Bruns A., Bruyn G.A., Collado P., De la Cruz L., Guillaume-Czitrom S., Herlin T. (2017). Preliminary Definitions for the Sonographic Features of Synovitis in Children. Arthritis Care Res..

[28] Vojinovic J., Magni-Manzoni S., Collado P., Windschall D., Ravagnani V., Hernandez Díaz C., Gonzales J., Malattia C., Tzaribachev N., Susic G. (2017). SAT0636 Ultrasonography Definitions for Synovitis Grading in Children: The Omeract Pediatric Ultrasound Task Force. Ann. Rheum. Dis..

[29] Sande N.K., Bøyesen P., Aga A.-B., Hammer H.B., Flatø B., Roth J., Lilleby V. (2021). Development and Reliability of a Novel Ultrasonographic Joint-Specific Scoring System for Synovitis with Reference Atlas for Patients with Juvenile Idiopathic Arthritis. RMD Open.

[30] Terslev L., Naredo E., Iagnocco A., Balint P.V., Wakefield R.J., Aegerter P., Aydin S.Z., Bachta A., Hammer H.B., Bruyn G.A.W. (2014). Defining Enthesitis in Spondyloarthritis by Ultrasound: Results of a Delphi Process and of a Reliability Reading Exercise. Arthritis Care Res..

[31] Wakefield R.J., Balint P.V., Szkudlarek M., Filippucci E., Backhaus M., D’Agostino M.-A., Sanchez E.N., Iagnocco A., Schmidt W.A., Bruyn G.A.W. (2005). Musculoskeletal Ultrasound Including Definitions for Ultrasonographic Pathology. J. Rheumatol..

[32] Collado P., Magni-Manzoni S., Steiner M., Ting T., Fernandez P.V., Malattia C., Rodriguez A., Bruyn G., Keen H., Terslev L. (2019). Fri0635 Ultrasound in the Assessment of Tenosynovitis in Juvenile Idiopathic Arthritis: Systematic Literature Review. Ann. Rheum. Dis..

[33] Collado P., Lanni S., Lucia O.D., Balint P., Guillaume S., Hernandez-Diaz C., Sande N.M.K., Magni-Manzoni S., Malattia C., Martire V. (2022). Pos1385 International Consensus for Ultrasound Definitions of Tenosynovitis in Juvenile Idiopathic Arthritis: Results of a Delphi Process. Ann. Rheum. Dis..

[34] Ventura-Ríos L., Faugier E., Barzola L., De la Cruz-Becerra L.B., Sánchez-Bringas G., García A.R., Maldonado R., Roth J., Hernández-Díaz C. (2018). Reliability of Ultrasonography to Detect Inflammatory Lesions and Structural Damage in Juvenile Idiopathic Arthritis. Pediatr. Rheumatol. Online J..

[35] Rossi-Semerano L., Breton S., Semerano L., Boubaya M., Ohanyan H., Bossert M., Boiu S., Chatelus E., Durand G., Jean S. (2021). Application of the OMERACT Synovitis Ultrasound Scoring System in Juvenile Idiopathic Arthritis: A Multicenter Reliability Exercise. Rheumatology.

[36] Magni-Manzoni S., Epis O., Ravelli A., Klersy C., Veisconti C., Lanni S., Muratore V., Sciré C.A., Rossi S., Montecucco C. (2009). Comparison of Clinical versus Ultrasound-Determined Synovitis in Juvenile Idiopathic Arthritis. Arthritis Rheum..

[37] Breton S., Jousse-Joulin S., Cangemi C., de Parscau L., Colin D., Bressolette L., Saraux A., Devauchelle-Pensec V. (2011). Comparison of Clinical and Ultrasonographic Evaluations for Peripheral Synovitis in Juvenile Idiopathic Arthritis. Semin. Arthritis Rheum..

[38] Rebollo-Polo M., Koujok K., Weisser C., Jurencak R., Bruns A., Roth J. (2011). Ultrasound Findings on Patients with Juvenile Idiopathic Arthritis in Clinical Remission. Arthritis Care Res..

[39] Haslam K.E., McCann L.J., Wyatt S., Wakefield R.J. (2010). The Detection of Subclinical Synovitis by Ultrasound in Oligoarticular Juvenile Idiopathic Arthritis: A Pilot Study. Rheumatology.

[40] Pascoli L., Wright S., McAllister C., Rooney M. (2010). Prospective Evaluation of Clinical and Ultrasound Findings in Ankle Disease in Juvenile Idiopathic Arthritis: Importance of Ankle Ultrasound. J. Rheumatol..

[41] Rooney M.E., McAllister C., Burns J.F.T. (2009). Ankle Disease in Juvenile Idiopathic Arthritis: Ultrasound Findings in Clinically Swollen Ankles. J. Rheumatol..

[42] Lanni S., Bovis F., Ravelli A., Viola S., Magnaguagno F., Pistorio A., Michele Magnano G., Martini A., Malattia C. (2016). Delineating the Application of Ultrasound in Detecting Synovial Abnormalities of the Subtalar Joint in Juvenile Idiopathic Arthritis. Arthritis Care Res..

[43] Della Paolera S., Pastore S., Zabotti A., Tommasini A., Taddio A. (2022). Ultrasonographic Assessment for Tenosynovitis in Juvenile Idiopathic Arthritis with Ankle Involvement: Diagnostic and Therapeutic Significance. Children.

[44] Licciardi F., Petraz M., Covizzi C., Santarelli F., Cirone C., Mulatero R., Robasto F., Dellepiane M., Martino S., Montin D. (2022). Discordance between Clinical and Ultrasound Examinations in Juvenile Idiopathic Arthritis: An Experimental Approach. Children.

[45] Jousse-Joulin S., Breton S., Cangemi C., Fenoll B., Bressolette L., de Parscau L., Saraux A., Devauchelle-Pensec V. (2011). Ultrasonography for Detecting Enthesitis in Juvenile Idiopathic Arthritis. Arthritis Care Res..

[46] Weiss P.F., Chauvin N.A., Klink A.J., Localio R., Feudtner C., Jaramillo D., Colbert R.A., Sherry D.D., Keren R. (2014). Detection of Enthesitis in Children with Enthesitis-Related Arthritis: Dolorimetry Compared to Ultrasonography. Arthritis Rheumatol..

[47] Shenoy S., Aggarwal A. (2016). Sonologic Enthesitis in Children with Enthesitis-Related Arthritis. Clin. Exp. Rheumatol..

[48] Ikeda K., Kambe N., Takei S., Nakano T., Inoue Y., Tomiita M., Oyake N., Satoh T., Yamatou T., Kubota T. (2014). Ultrasonographic Assessment Reveals Detailed Distribution of Synovial Inflammation in Blau Syndrome. Arthritis Res. Ther..

[49] Aptel S., Lecocq-Teixeira S., Olivier P., Regent D., Teixeira P.G., Blum A. (2016). Multimodality Evaluation of Musculoskeletal Sarcoidosis: Imaging Findings and Literature Review. Diagn. Interv. Imaging.

[50] Osman H.T., Mostafa N., Marzouk H., Sabry N., Abdou M., Khalifa I. (2022). Ultrasound Hand and Wrist Findings in Children with Systemic Lupus Erythematosus. Curr. Rheumatol. Rev..

[51] Salliot C., Denis A., Dernis E., Andre V., Perdriger A., Albert J.-D., Mammou Mraghni S., Griffoul-Espitalier I., Hamidou M., Le Goff B. (2018). Ultrasonography and Detection of Subclinical Joints and Tendons Involvements in Systemic Lupus Erythematosus (SLE) Patients: A Cross-Sectional Multicenter Study. Jt. Bone Spine.

[52] 2019 American College of Rheumatology/Arthritis Foundation Guideline for the Treatment of Juvenile Idiopathic Arthritis: Therapeutic Approaches for Non-Systemic Polyarthritis, Sacroiliitis, and Enthesitis—PubMed. https://pubmed.ncbi.nlm.nih.gov/31021516/.

[53] Young C.M., Horst D.M., Murakami J.W., Shiels W.E. (2015). Ultrasound-Guided Corticosteroid Injection of the Subtalar Joint for Treatment of Juvenile Idiopathic Arthritis. Pediatr. Radiol..

[54] Laurell L., Court-Payen M., Nielsen S., Zak M., Fasth A. (2012). Ultrasonography and Color Doppler in Juvenile Idiopathic Arthritis: Diagnosis and Follow-up of Ultrasound-Guided Steroid Injection in the Wrist Region. A Descriptive Interventional Study. Pediatr. Rheumatol. Online J..

[55] Laurell L., Court-Payen M., Nielsen S., Zak M., Boesen M., Fasth A. (2011). Ultrasonography and Color Doppler in Juvenile Idiopathic Arthritis: Diagnosis and Follow-up of Ultrasound-Guided Steroid Injection in the Ankle Region. A Descriptive Interventional Study. Pediatr. Rheumatol. Online J..

[56] Collado P., Naredo E., Calvo C., Gamir M.L., Calvo I., García M.L., Merino R., Graña J., Bustabab S., Garrido J. (2013). Reduced Joint Assessment vs Comprehensive Assessment for Ultrasound Detection of Synovitis in Juvenile Idiopathic Arthritis. Rheumatology.

[57] Vega-Fernandez P., Ting T.V., Oberle E.J., McCracken C., Figueroa J., Altaye M., Cassedy A., Kaeley G.S., Roth J. (2021). CARRA Musculoskeletal Ultrasound Workgroup The MUSICAL Pediatric Ultrasound Examination—A Comprehensive, Reliable, Time Efficient Assessment of Synovitis. Arthritis Care Res..

[58] Lazarevic D.S., Vojinovic J., Malattia C., Rossi-Semerano L., Sozeri B., Tsinti M., Lanni S., Host C., Windschall D., Snipaitiene A. (2022). P204—Internal Consistency and Interrater Reliability in Musculoskeletal Ultrasound in Children. Pediatr. Rheumatol..

[59] De Lucia O., Ravagnani V., Pregnolato F., Hila A., Pontikaki I., Gattinara M., Romano M., Gerloni V., Pieropan S., Murgo A. (2018). Baseline Ultrasound Examination as Possible Predictor of Relapse in Patients Affected by Juvenile Idiopathic Arthritis (JIA). Ann. Rheum. Dis..

[60] Miotto E Silva V.B., Mitraud S.d.A.V., Furtado R.N.V., Natour J., Len C.A., Terreri M.T.d.S.E. (2017). Patients with Juvenile Idiopathic Arthritis in Clinical Remission with Positive Power Doppler Signal in Joint Ultrasonography Have an Increased Rate of Clinical Flare: A Prospective Study. Pediatr. Rheumatol. Online J..

[61] Haavardsholm E.A., Aga A.-B., Olsen I.C., Lillegraven S., Hammer H.B., Uhlig T., Fremstad H., Madland T.M., Lexberg Å.S., Haukeland H. (2016). Ultrasound in Management of Rheumatoid Arthritis: ARCTIC Randomised Controlled Strategy Trial. BMJ.

[62] Magni-Manzoni S., Scirè C.A., Ravelli A., Klersy C., Rossi S., Muratore V., Visconti C., Lanni S., Merli P., Montecucco C. (2013). Ultrasound-Detected Synovial Abnormalities Are Frequent in Clinically Inactive Juvenile Idiopathic Arthritis, but Do Not Predict a Flare of Synovitis. Ann. Rheum. Dis..

[63] Zhao Y., Rascoff N.E., Iyer R.S., Thapa M., Reichley L., Oron A.P., Wallace C.A. (2018). Flares of Disease in Children with Clinically Inactive Juvenile Idiopathic Arthritis Were Not Correlated with Ultrasound Findings. J. Rheumatol..

[64] Ravelli A., Consolaro A., Horneff G., Laxer R.M., Lovell D.J., Wulffraat N.M., Akikusa J.D., Al-Mayouf S.M., Antón J., Avcin T. (2018). Treating Juvenile Idiopathic Arthritis to Target: Recommendations of an International Task Force. Ann. Rheum. Dis..

[65] Wallace C.A., Ruperto N., Giannini E. (2004). Childhood Arthritis and Rheumatology Research Alliance; Pediatric Rheumatology International Trials Organization; Pediatric Rheumatology Collaborative Study Group Preliminary Criteria for Clinical Remission for Select Categories of Juvenile Idiopathic Arthritis. J. Rheumatol..

[66] D’Agostino M.-A., Wakefield R.J., Berner-Hammer H., Vittecoq O., Filippou G., Balint P., Möller I., Iagnocco A., Naredo E., Østergaard M. (2016). Value of Ultrasonography as a Marker of Early Response to Abatacept in Patients with Rheumatoid Arthritis and an Inadequate Response to Methotrexate: Results from the APPRAISE Study. Ann. Rheum. Dis..

[67] do Prado A.D., Bisi M.C., Piovesan D.M., Bredemeier M., Batista T.S., Petersen L., Bauer M.E., da Silveira I.G., Mendonça J.A., Staub H.L. (2016). Ultrasound Power Doppler Synovitis Is Associated with Plasma IL-6 in Established Rheumatoid Arthritis. Cytokine.

[68] Foell D., Wulffraat N., Wedderburn L.R., Wittkowski H., Frosch M., Gerss J., Stanevicha V., Mihaylova D., Ferriani V., Tsakalidou F.K. (2010). Methotrexate Withdrawal at 6 vs 12 Months in Juvenile Idiopathic Arthritis in Remission: A Randomized Clinical Trial. JAMA.

[69] Anink J., Van Suijlekom-Smit L.W.A., Otten M.H., Prince F.H.M., van Rossum M.A.J., Dolman K.M., Hoppenreijs E.P.A.H., ten Cate R., Ursu S., Wedderburn L.R. (2015). MRP8/14 Serum Levels as a Predictor of Response to Starting and Stopping Anti-TNF Treatment in Juvenile Idiopathic Arthritis. Arthritis Res. Ther..

[70] Eich G.F., Hallé F., Hodler J., Seger R., Willi U.V. (1994). Juvenile Chronic Arthritis: Imaging of the Knees and Hips before and after Intraarticular Steroid Injection. Pediatr. Radiol..

[71] Gohar F., Windschall D. (2021). The New Role of Musculoskeletal Ultrasound in the Treat-to-Target Management of Juvenile Idiopathic Arthritis. Rheumatology.

[72] Colebatch-Bourn A.N., Edwards C.J., Collado P., D’Agostino M.-A., Hemke R., Jousse-Joulin S., Maas M., Martini A., Naredo E., Østergaard M. (2015). EULAR-PReS Points to Consider for the Use of Imaging in the Diagnosis and Management of Juvenile Idiopathic Arthritis in Clinical Practice. Ann. Rheum. Dis..

[73] Marteau P., Adamsbaum C., Rossi-Semerano L., De Bandt M., Lemelle I., Deslandre C., Tran T.A., Lohse A., Solau-Gervais E., Sordet C. (2018). Conventional Radiography in Juvenile Idiopathic Arthritis: Joint Recommendations from the French Societies for Rheumatology, Radiology and Paediatric Rheumatology. Eur. Radiol..

[74] Hemke R., Herregods N., Jaremko J.L., Åström G., Avenarius D., Becce F., Bielecki D.K., Boesen M., Dalili D., Giraudo C. (2020). Imaging Assessment of Children Presenting with Suspected or Known Juvenile Idiopathic Arthritis: ESSR-ESPR Points to Consider. Eur. Radiol..

[75] Malattia C., Damasio M.B., Magnaguagno F., Pistorio A., Valle M., Martinoli C., Viola S., Buoncompagni A., Loy A., Ravelli A. (2008). Magnetic Resonance Imaging, Ultrasonography, and Conventional Radiography in the Assessment of Bone Erosions in Juvenile Idiopathic Arthritis. Arthritis Rheum..

[76] Pradsgaard D.Ø., Spannow A.H., Heuck C., Herlin T. (2013). Decreased Cartilage Thickness in Juvenile Idiopathic Arthritis Assessed by Ultrasonography. J. Rheumatol..

[77] Mitra S., Samui P.P., Samanta M., Mondal R.K., Hazra A., Mandal K., Sabui T.K. (2019). Ultrasound Detected Changes in Joint Cartilage Thickness in Juvenile Idiopathic Arthritis. Int. J. Rheum. Dis..

